# Down-regulation of the sucrose transporters HvSUT1 and HvSUT2 affects sucrose homeostasis along its delivery path in barley grains

**DOI:** 10.1093/jxb/erx266

**Published:** 2017-08-24

**Authors:** Volodymyr Radchuk, David Riewe, Manuela Peukert, Andrea Matros, Marc Strickert, Ruslana Radchuk, Diana Weier, Hans-Henning Steinbiß, Nese Sreenivasulu, Winfriede Weschke, Hans Weber

**Affiliations:** 1 Leibniz Institut für Pflanzengenetik und Kulturpflanzenforschung, Stadt Seeland OT Gatersleben, Germany; 2 Computational Intelligence—FB12 Informatik, Philipps University, Marburg, Germany; 3 Max-Planck-Institut für Züchtungsforschung, Köln, Germany

**Keywords:** Assimilate transport, metabolite profiling, starch synthesis, sucrose transporter, transcript profiling, vacuole

## Abstract

Sucrose transport and partitioning are crucial for seed filling. While many plasma-membrane-localised sucrose transporters (SUT1 family members) have been analysed in seeds, the functions of vacuolar SUT2 members are still obscure. In barley grains, expression of *HvSUT1* and *HvSUT2* overlap temporally and spatially, suggesting concerted functions to regulate sucrose homeostasis. Using *HvSUT2*-RNAi plants, we found that grains were also deficient in *HvSUT1* expression and seemingly sucrose-limited during mid-to-late grain filling. Transgenic endosperms accumulated less starch and dry weight, although overall sucrose and hexose contents were higher. Comprehensive transcript and metabolite profiling revealed that genes related to glycolysis, the tricarboxylic acid cycle, starch and amino acid synthesis, grain maturation, and abscisic acid signalling were down-regulated together with most glycolytic intermediates and amino acids. Sucrose was increased along the sucrose delivery route in the nucellar projection, the endosperm transfer cells, and the starchy endosperm, indicating that suppressed transporter activity diminished sucrose efflux from vacuoles, which generated sugar deficiency in the cytoplasm. Thus, endosperm vacuoles may buffer sucrose concentrations to regulate homeostasis at grain filling. Transcriptional changes revealed that limited endosperm sucrose initiated sugar starvation responses, such as sugar recycling from starch, hemicelluloses and celluloses together with vacuolar protein degradation, thereby supporting formation of nucleotide sugars. Barley endosperm cells can thus suppress certain pathways to retrieve resources to maintain essential cell functions.

## Teaser

RNAi-repression of vacuolar HvSUT2 in transgenic barley demonstrates its indispensable role for proper grain filling and for the control of sucrose homeostasis in concert with the plasma-membrane localised HvSUT1.

## Introduction

In many higher plants, sucrose is the main carbohydrate for transport and storage. It is partitioned between different organs by membrane-integrated sucrose/H^+^ co-transport proteins (SUTs), in an energy-dependent manner. SUT-mediated sucrose transport and partitioning is of crucial importance for plant growth and development as well as seed filling.

SUTs are encoded by small gene families divided into four or five major clades (Kühn and Grof, 2010; Sauer, 2007). SUT1 members have high substrate affinities and exhibit organ- and tissue-specific expression. They are frequently expressed in leaf vascular tissues and are essential for phloem loading (Riesmeier *et al.*, 1994; Gottwald *et al.*, 2000; Slewinski *et al.*, 2009). Some also function in sucrose transport to sink organs such as seeds (Weber *et al.*, 1997; Tegeder *et al.*, 1999). SUT1 members are highly expressed in developing grains of different cereal crops. In rice, *OsSUT1* is expressed at similar levels from early to late seed filling (Furbank *et al.*, 2001). *HvSUT1* of barley is most highly expressed during early to mid-grain filling. Transcripts of *OsSUT1* and *HvSUT1* are localised mainly in the maternal nucellar projection, in the inner integument, and in the filial transfer cells that separate the endosperm cavity from the endosperm (Weschke *et al.*, 2000; Furbank *et al.*, 2001).

Some members of the SUT4 clade such as Arabidopsis AtSUT4, barley HvSUT2, rice OsSUT2, wheat TaSUT2, *Lotus japonicus* LjSUT4, and poplar PtaSUT4 are localised in vacuolar membranes, as demonstrated by proteomic and/or GFP-fusion analyses (Endler *et al.*, 2006; Reinders *et al.*, 2008; Payyavula *et al.*, 2011). Patch clamp experiments reveal that HvSUT2, LjSUC4, and AtSUC4 export sucrose with low affinity from vacuoles into the cytoplasm (Reinders *et al.*, 2008; Schulz *et al.*, 2011). Vacuolar sucrose transporters are broadly expressed, indicating the requirement for sucrose transfer across the tonoplast in many cell types. Barley *HvSUT2* is expressed in leaves, roots, pericarp, and endosperm of developing grains (Weschke *et al.*, 2000). *AtSUT4* is expressed in companion and leaf mesophyll cells (Schulze *et al.*, 2003; Endler *et al.*, 2006). *PtaSUT4*-RNAi poplar plants show an increased leaf-to-stem biomass ratio, indicating a relationship between vacuolar sucrose transport and biomass partitioning (Payyavula *et al.*, 2011). The *OsSUT2* mutation affects sucrose efflux from the vacuole and reduces seed production and root growth, which indicates that sucrose transport to sink organs is impaired (Eom *et al.*, 2011).

Seed filling depends on assimilate supply. The cellular pathway of sucrose transport into developing wheat or barley grains is restricted to vascular bundles located at the bottom of the crease and extending across the whole grain length. The crease vein constitutes the site of phloem unloading, and photoassimilates are transported symplastically inwards and are unloaded into the endosperm cavity (Thorne, 1985). Cellular sites of efflux are membranes of the nucellar projection (NP) cells proximal to the endosperm, which develop wall ingrowths to increase the membrane surface (Thiel *et al.*, 2012). Sucrose is imported by SUT1-like transporters into epidermal and/or endosperm transfer cells (ETCs) by energy-dependent H^+^-co-transport and is subsequently degraded by sucrose synthase in endosperm cells as a first step towards starch biosynthesis (Weber *et al.*, 1997, 2005; Tegeder *et al.*, 1999; Weschke *et al.*, 2000). Rapid induction of *HvSUT1* expression in the ETCs at 6 to 7 d after fertilisation (DAF) coincides with increasing levels of sucrose and sucrose synthase mRNA and activity, and occurs immediately before the onset of endosperm starch accumulation. Similar to *HvSUT1*, wheat *TaSUT1* is highly expressed during mid-seed filling (Weschke *et al.*, 2000; Weichert *et al.*, 2010) and is important for grain filling. Antisense-suppression of the rice-homolog *OsSUT1* strongly reduces grain starch and generates wrinkled phenotypes (Scofield *et al.*, 2002).

The wheat tonoplast-localised *TaSUT2* is highly expressed in the flag leaf blade before anthesis and during early grain filling, suggesting intracellular partitioning in leaves to allocate assimilates from leaves to grains. TaSUT1 and TaSUT2 are functionally and temporally co-ordinated during seed development (Deol *et al.*, 2013). Whereas TaSUT2 might control cytosolic sucrose homeostasis in leaves, TaSUT1 secures sucrose loading in the endosperm. Tonoplast *HvSUT2* from barley and *OsSUT2* from rice (Sun *et al.*, 2008) are highly expressed in source leaves, consistent with the fact that cereal leaves transiently store sucrose rather than starch in vacuoles (Martinoia *et al.*, 2007). *HvSUT2* is strongly expressed in several grain tissues, including the endosperm, with very similar profiles to *HvSUT1* (Weschke *et al.*, 2000).

Whereas the functions of SUT1 in grain filling in cereals have been analysed extensively, the significance of vacuolar SUTs is more obscure, especially related to grain filling and development. Plant vacuoles are potentially important for energy homeostasis, storage of nutrients, cellular pressure, detoxification, and environmental interactions. Vacuoles are transient storage pools for metabolites produced in excess, allowing them to be released to the cytoplasm when required. In particular, seed storage cells contain large central vacuoles, which could function as transient repositories for sugars and amino acids (Neuhaus, 2007).

In this study, the role of sucrose transporters in assimilate allocation in the developing grains of barley was analysed. It was found that RNAi-mediated repression of *HvSUT2* also resulted in down-regulation of *HvSUT1* and led to accumulation of sucrose along the sucrose delivery route in the nucellar projection, the endosperm transfer cells, and the starchy endosperm, although grains become apparently sucrose-limited. The results suggest that both HvSUT2 and HvSUT1 control sucrose homeostasis during grain filling. Their deficiency affects maturation and certain biosynthetic pathways, and initiates sugar starvation-induced salvage of resources.

## Material and methods

### Plant material

Plants of wild-type and transgenic barley (*Hordeum vulgare* L. cv. Barke) were grown under standard greenhouse conditions at 18 °C with 16 h light. Sample preparation was performed as described by Weschke *et al.* (2000).

### Cloning of *SUT*-sequences of barley and sequence analysis

To search for *SUT*-cDNAs and genes, BLASTN screening was performed on a barley cDNA-library (Matsumoto *et al.*, 2011) and full-length genome sequence (Mascher *et al.*, 2017) using rice-cDNAs as queries (Ibraheem *et al.*, 2010). Full-length cDNAs of *HvSUT3* and *HvSUT4* were RT-PCR-amplified from total RNA from young caryopses using gene-specific primers (Supplementary Table S1 at *JXB* online) derived from predicted cDNAs. The *HvSUT1* promoter region was PCR-amplified from genomic DNA using specific primers (Supplementary Table S1). Sequence data were processed using Lasergene software (www.dnastar.com) and ClustalW software (www.clustal.org) to build the phylogenetic tree.

### RNA isolation and qRT-PCR

Total RNA was extracted from dissected pericarp and endosperm fractions as described previously (Radchuk *et al.*, 2012). A total of 5 µg RNA was used for reverse transcription by SuperScript III reverse-transriptase (Invitrogen) with oligo(dT) primer. The Resulting cDNAs were used as a template for quantitative RT-PCR analyses with three replications (Radchuk *et al.*, 2012). Data were analysed using SDS 2.2.1 software (Applied Biosystems). The highest relative normalised expression was taken as 100% and the expression of other genes recalculated on this basis.

### Vector construction and plant transformation

The RNAi construct p1SUT2i for barley stable transformation consists of the *HvSUT1* promoter (1445 nt 5′-upstream of the ATG start codon of the *HvSUT1* gene), the sense-fragment of *HvSUT2* (470 nt), the first intron of potato GA20-oxidase (199 nt), and the antisense-fragment of *HvSUT2* (470 nt). Appropriate DNA fragments were PCR-amplified and cloned into the modified pUC19 vector. Primers are listed in Supplementary Table S1. The cassette was cut from pUC19 with *Pst* I and cloned into the intermediate vector pBluescript. By cutting out with *Not* I/*Hind*III restriction enzymes, the cassette was introduced into the corresponding sites of the binary vector pWVec8. RNAi-lines were generated by *Agrobacterium tumefaciens*-mediated transformation of immature embryos from *H. vulgare* cv. Golden Promise as described previously (Radchuk *et al.*, 2006). Transgenic lines S12i, S20i, and S80i were chosen for analysis.

To characterise the intracellular localisation of HvSUT1 and HvSUT2, open reading frames of corresponding cDNAs were fused to green fluorescence protein (GFP) and cloned under the control of the *CaMV35S* promoter to create constructs: *pHvSUT1-GFP* and *pHvSUT2-GFP*. Arabidopsis protoplasts were isolated from suspension culture and transiently transformed as described previously (Radchuk *et al.*, 2012). GFP-signals were measured *in vivo* with a Zeiss LSM510 META confocal laser scanning microscope (www.zeiss.de).

### *In situ* hybridisation

For *in situ* hybridisation, grains were fixed in 50% (v/v) ethanol, 5% (v/v) acetic acid, and 3.7% (w/v) formaldehyde overnight at 40 °C, dehydrated, and embedded in paraffin. Cross-sections (12μm) were de-waxed, rehydrated, and treated with 2μg ml^−1^ proteinase K for 30min at 37 °C. Tissue sections were dehydrated and dried before applying the hybridisation solution. Hybridisations were performed with 1 ng μl^−1^ digoxigenin-labelled sense and antisense RNA probes for *HvSUT1* and *HvSUT2*-cDNA synthesised using T3 or T7 RNA polymerase (Roche, Germany). Hybridisation signals were detected by alkaline phosphatase-conjugated anti-digoxigenin antibody and visualised with 4-nitroblue tetrazolium-chloride and 5-bromo-4-chloro-3-indolyl phosphate (www.roche.de).

### Determination of sucrose, starch, amino acids, and total carbon and nitrogen

Sucrose and starch were determined by enzyme-coupled assays (Rolletschek *et al.*, 2002). Relative contents of total carbon and nitrogen in dried, powdered samples were measured using an elemental analyser (Vario EL; Elementar Analysensysteme, www.elementar.de). Statistical analysis was performed using Student’s *t*-test, Sigma Stat software (SPSS; www.systat.de). Analysis of amino acids was performed using a AccQ®Tag Ultra column (2.1 × 100 mm) using a Waters Acquity UPLC® system (www.waters.com), equipped with a fluorescence detector as described previously (Thiel *et al.*, 2009). Analysis of polar metabolites was performed using a LECO Pegasus HT mass spectrometer coupled to an Agilent 7890 gas chromatograph and a Gerstel MPS-XL auto-sampler as described previously (Riewe *et al.*, 2012), yielding 79 assigned metabolites (Supplementary Table S3). Multiple *t*-test correction was performed (Benjamini and Hochberg, 1995).

Micro-dissected tissues were prepared as described by Kohl *et al.* (2015). Four caryopses from ears at 16, 20, and 24 DAF were isolated from S20i and wild-type plants and cryo-sectioned (30 µm). Sections from the NP, ETCs, and the centre of the starch endosperm were prepared to produce eight biological repetitions, which contained at least eight dissects each. Sucrose was extracted and measured using GC-MS as described above.

### cDNA-array and data analysis

Probe preparation, hybridisation, and processing of the 12K barley seed cDNA array was performed as described by Sreenivasulu *et al.* (2006) with manually up-graded gene annotations. Images of hybridised nylon membranes were subjected to automatic spot detection using the MATLAB program and the signal intensities of 11 787 genes from the double spots were scored to enable assessment of two technical replications. Additionally, two biological repetitions were performed using RNA from independently grown plants to check the biological reproducibility. Quantile-normalisation was carried out on the complete data set. Fold-changes between wild-type and transgenic samples were calculated from two technical and two biological replicates. *P*-values were calculated based on moderated *t*-tests to detect false positives. Only 2-fold and higher expression differences for genes that were significantly differentially expressed between transgenic and wild-type lines were selected for further analyses. A detailed set of normalised values, fold-difference, and *P*-values of differentially expressed genes is provided in Supplementary Table S2.

### Mass spectrometry imaging (MSI)

Matrix-assisted laser desorption ionization (MALDI) MSI visualises spatial distributions of molecular ions (Caprioli *et al.*, 1997) and has been applied on plants (Kaspar *et al.*, 2011; Matros and Mock, 2013), including developing and germinating barley grains (Peukert *et al.*, 2014; Gorzolka *et al.*, 2016). Generally, MSI creates an array of mass spectra, with each spot representing its own *m/z* profile. Gradients of ion abundances are visualised across samples by two-dimensional reconstruction of ion chromatograms using colour coding to display intensity distributions.

Isolated grains of S20i and wild-type plants at 14 and 20 DAF were snap-frozen in liquid nitrogen and stored at –80 °C. Frozen grains were fixed to sample holders of a cryotome (–20 °C) using optimal cutting temperature compound (OCT™, Tissue-Tek® Sakura Finetek Europe B.V.). Cross-sections (30 µm) were cut, thaw-mounted onto indium tin oxide (ITO)-coated glass slides (Bruker Daltonics) and dried for 30 min. Prior to matrix application, images were captured using a stereomicroscope (Leica MZ6) connected to a digital camera (AxioCam ICc1, Zeiss). The matrix [2,5-dihydroxybenzoic acid (Sigma-Aldrich) diluted to 30 mg ml^–1^ in 50% v/v methanol and 0.2% (w/v) trifluoroacetic acid] was applied using sensor-controlled vibrational vaporisation using an ImagePrep device (www.bruker.com). MSI measurements were performed with three-fold (14 DAF) or two-fold (20 DAF) repetition (Peukert *et al.*, 2014). The laser raster (resolution) was set between 20 and 35 µm depending on the size of the measured section area: for the transfer region (14 DAF) the laser raster was 20 µm and for whole-grain sections (20 DAF) the laser raster was 35 µm. Acquisition of laser raster spots was carried out randomly and simultaneously for S20i and wild-type samples to eliminate bias. Mass calibration was performed with a polyethylene glycol mixture: 1:1 PEG200 and 600, diluted 1:300 in 30% v/v acetonitrile and 0.1% w/v trifluoroacetic acid. Data sets were loaded into SCiLS Lab software (www.SCiLS.de) for visualisation of *m/z* values and statistical analysis. Distribution patterns of selected *m/z* values were displayed as single-ion intensity maps by two-dimensional reconstruction onto section images. Signal intensities were evaluated statistically for differences between tissues and between S20i and wild-type samples. Tissues (NP and ETC) were manually selected based on microscopy. An Anderson–Darling test (implemented in SCiLS Lab software) was applied to test normal distributions. Since only some peaks were normally distributed, significances were calculated by a Wilcoxon test.

## Results

### Identification of sucrose transporters in barley

**Fig. 1.**
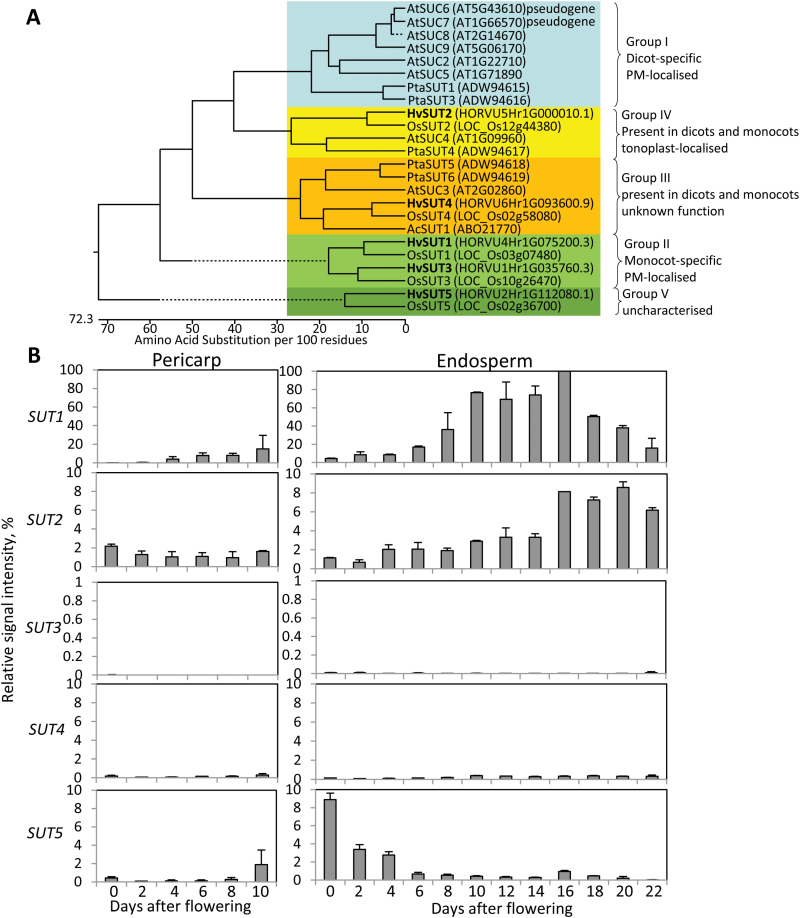
(A) Phylogenetic tree of selected SUT proteins, constructed using the neighbour-joining-method. Genbank numbers are given in brackets. Barley sequences are shown in bolt. Ac, *Ananas comosus*; At, *Arabidopsis thaliana*; Hv, *Hordeum vulgare*; Os, *Oryza sativa*. (B) Transcript profiles of *HvSUT1*–*HvSUT5* in separated pericarp and endosperm fractions measured by quantitative RT-PCR. Values are means ±SD, *n*=3. (This figure is available in colour at *JXB* online.)

To characterise *SUT* gene family members in barley, a full genome sequence (Mascher *et al.*, 2017) was screened using rice cDNAs as queries (Ibraheem *et al.*, 2010), yielding five candidates. Two sequences encoded the previously described *HvSUT1* (gene ID *HORVU4Hr1G075200.3*) and *HvSUT2* (*HORVU5Hr1G000010.1*) genes (Weschke *et al.*, 2000; Endler *et al.*, 2006). The other three sequences, named *HvSUT3* (*HORVU1Hr1G035760.3*), *HvSUT4* (*HORVU6Hr1G093600.9*), and *HvSUT5* (*HORVU2Hr1G112080.1*), were highly similar to the corresponding rice counterparts (Fig. 1A). Full-length open reading frames were cloned by RT-PCR and re-sequenced.

A phylogenetic tree was constructed by the neighbour-joining method using predicted SUT protein sequences from barley, rice, Arabidopsis, and poplar (Fig. 1A). The phylogeny showed that HvSUT1 and HvSUT3 grouped with clade II of the monocot-specific plasma-membrane-type transporters together with their rice counterparts. HvSUT2 clustered together with tonoplast-localised OsSUT2, AtSUC4, and PtaSUT4 (Endler *et al.*, 2006; Eom *et al.*, 2011; Payyavula *et al.*, 2011; Schneider *et al.*, 2012). HvSUT5 clustered together with OsSUT5 to clade V of monocot-specific but as yet uncharacterised SUTs. HvSUT4 is similar to OsSUT4 belonging to clade III of both monocot and dicot SUTs.

### Gene expression pattern of barley sucrose transporters

Expression of barley *SUT* genes was analysed in developing grains by quantitative RT-PCR (Fig. 1B). *HvSUT1* transcript levels were low in the pericarp and endosperm fractions between 0 and 4 d after fertilisation (DAF), increased from 6 to 14 DAF, the beginning of grain filling, and decreased at late grain filling (18 to 25 DAF). *HvSUT1* was expressed 10- to 100-fold higher in the endosperm compared to the other *HvSUT* genes. *HvSUT2* mRNA was weakly expressed throughout pericarp development and in early developing endosperm. However, transcript abundances increased at the beginning of storage accumulation until late grain filling (16 to 25 DAF). Both *HvSUT3* and *HvSUT4* transcripts were barely detectable in developing grains. *HvSUT5* expression was highest at anthesis in the embryo sac fraction, declining rapidly with the onset of seed development. Expression profiles indicated that only HvSUT1 and HvSUT2 were important during grain filling.

**Fig. 2.**
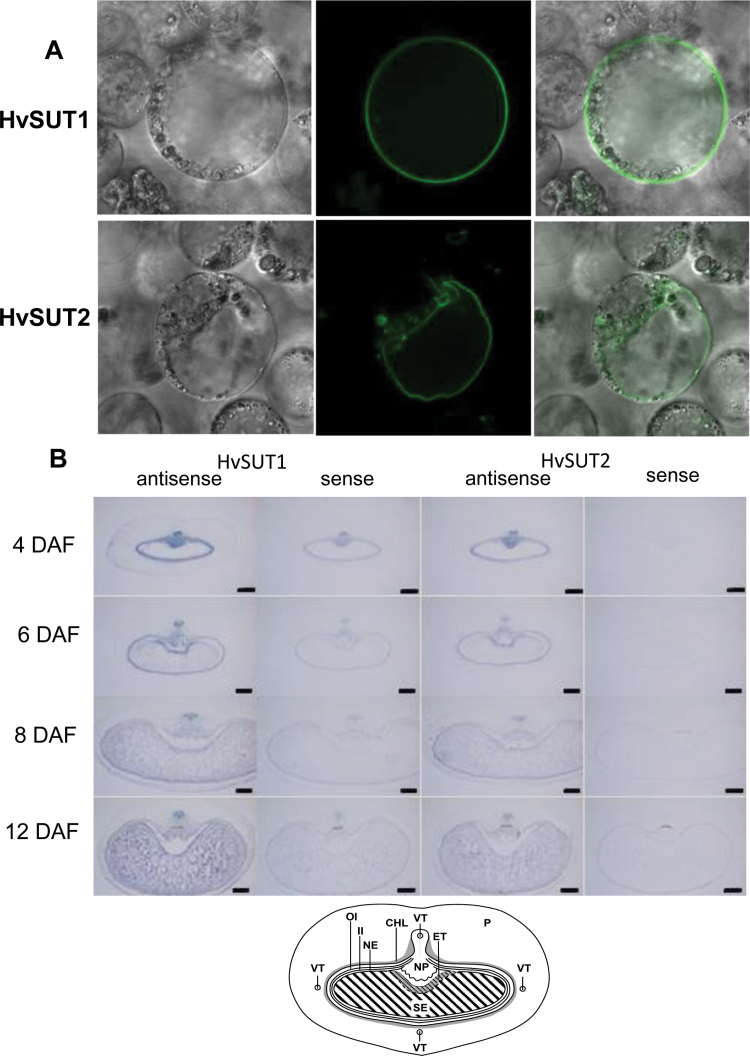
(A) Sub-cellular localisation of HvSUT1 and HvSUT2 in Arabidopsis protoplasts transiently transformed with pHvSUT1-GFP and pHvSUT2-GFP-fusion constructs under control of the cauliflower mosaic virus 35S-promoter. (B) Analysis of tissue-specific expression of *HvSUT1* and *HvSUT2*, performed by *in situ* hybridisation of cross-section of barley grains at 4, 6, 8, and 12 d after fertilisation (DAF) using single-stranded antisense-RNA along with sense controls. Scale bars =200 µm. The diagram below shows the tissue organisation of barley grains: CHL, chlorenchyma; ET, endosperm transfer cells; II, inner integument; NE, nucellar epidermis; OI, outer integument; P, pericarp; SE, starchy endosperm; VT, vascular tissue.

To verify sub-cellular localisation, protoplasts from Arabidopsis cell suspensions were transiently transformed with *HvSUT1-GFP* and *HvSUT2-GFP* fusion genes controlled by the cauliflower mosaic virus *35S* promoter. While HvSUT1-specific GFP fluorescence was localised at plasma-membranes (Fig. 2A, upper panel), HvSUT2-GFP-specific signals were detected at the large central and at smaller vacuoles (Fig. 2A, lower panel).

### Tissue-specific expression of *HvSUT1* and *HvSUT2* in developing grains

During grain development, assimilates are unloaded from vascular bundles through the NP into the apoplast and subsequently taken up by ETCs (Weschke *et al.*, 2000; Thiel *et al.*, 2008). Tissue-specific expression of *HvSUT1* and *HvSUT2* was analysed by *in-situ* hybridisation of cross-sections of barley grains at 4, 6, 8, and 12 DAF (Fig. 2B), using single-stranded antisense RNA along with sense controls. The distribution patterns of hybridisation signals were very similar, with stronger intensities for *HvSUT1*. In caryopses at 4 DAF, both *HvSUT1* and *HvSUT2* signals were detected in the NP, the chlorenchyma of the maternal pericarp, and the cells adjacent to vascular bundles, with weak signals in the pericarp. In ETCs, transcript-specific signals were detected at 4 and 6 DAF. During starch accumulation, specific signals were visible in starchy endosperm, with similar patterns for *HvSUT1* and *HvSUT2*.

The results indicated that *HvSUT1* and *HvSUT2* were expressed in tissues along the assimilate transfer path from the vascular bundle and the NP to ETCs and in starchy endosperm cells during grain filling. Gene expression patterns overlap temporally and spatially despite different sub-cellular localisation.

### Growth phenotype of transgenic plants with RNAi-mediated *HvSUT2* down-regulation

To analyse functions of the vacuolar HvSUT2 in sugar allocation, 30 transgenic plant lines with RNAi-mediated *HvSUT2* repression were generated via *Agrobacterium*-mediated transformation. The RNAi-construct was driven by the *HvSUT1* promoter because of its high activity (Fig. 1B) and co-expression with *HvSUT2* in the same grain cell types. Independent transformed plants were analysed for copy number by Southern gel blot analysis and *HvSUT2* repression. Three homozygous lines were selected and analysed, S12i, S20i, and S80i, containing one *HvSUT2*-RNAi copy and displaying decreased *HvSUT2* expression.

**Fig. 3.**
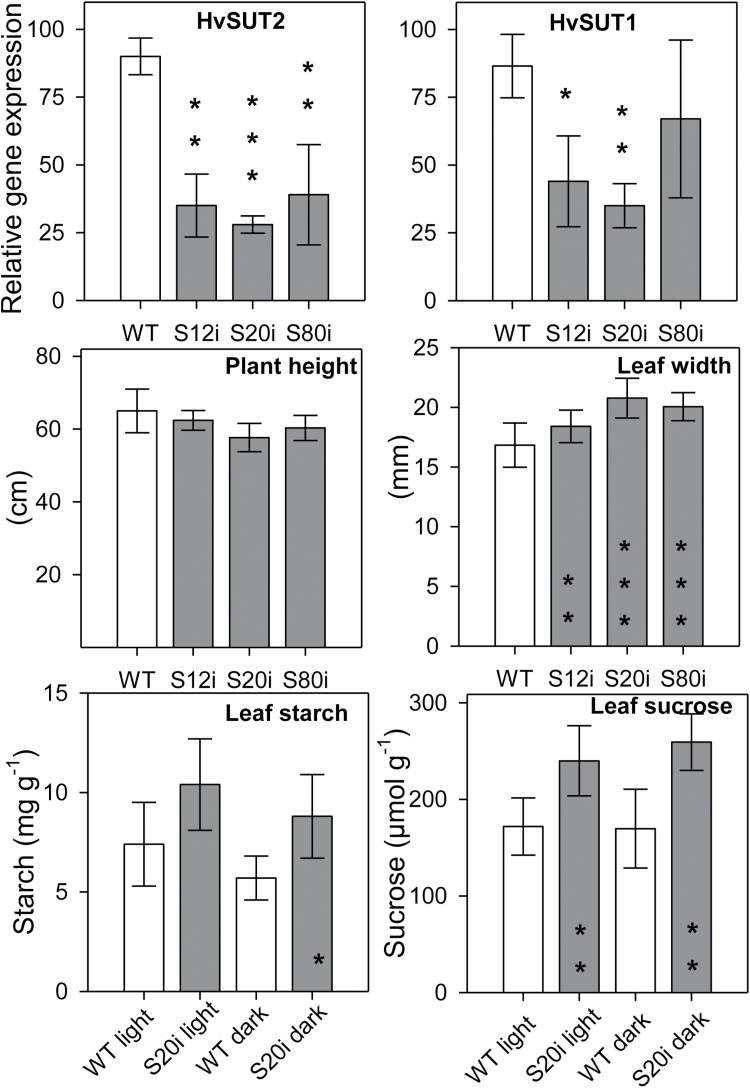
HvSUT1 and HvSUT2 expression in source leaves (means ±SD, *n*=3), plant height, and leaf width (means ±SD, *n*=10), leaf starch and sucrose (means ±SD, *n*=3) of HvSUT2-RNAi and wild-type plants. Significant differences were determined by *t*-tests: *P<0.05, **P<0.01, ***P<0.001.

In flag leaves at 10 DAF, *HvSUT2* expression was decreased by 50–60% in all three lines compared to the wild-type. Surprisingly, *HvSUT1* expression was also reduced, although to a lesser degree (Fig. 3). Whilst we cannot exclude the co-repression of *HvSUT1* by the *HvSUT2*-RNAi construct, the possibility of this is rather low due to weak identity (58.1%) between the *HvSUT2* fragment used for the RNAi construct and the corresponding cDNA fragment of *HvSUT1* (Supplementary Fig. S1). Instead, we consider this to be a consequence of the concerted action of HvSUT1 and HvSUT2, in which changes in abundance/activity of one transporter requires adjustments in the other. Plant architecture and height were not significantly changed. However, leaves of transgenic lines were significantly broader (Fig. 3). Sucrose and starch contents were significantly higher in transgenic leaves measured at midday and at the end of the night during the grain filling stage (Fig. 3).

### Phenotypic changes in the grains of transgenic plants

**Fig. 4.**
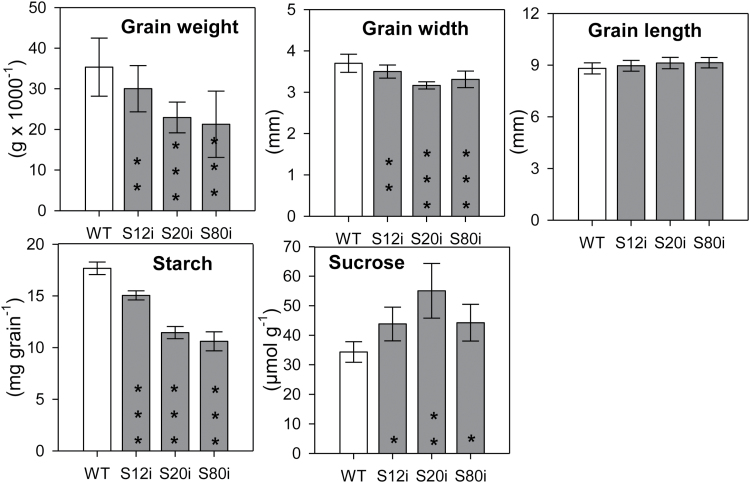
Characterisation of mature grains of three transgenic HvSUT2-RNAi lines compared to the wild-type. Grain weight, width, and length are means ±SD, *n*=200; grain starch and sucrose are means ±SD, *n*=3. Significant differences were determined by *t*-tests: *P<0.05, **P<0.01, ***P<0.001.

For the three selected transgenic lines, grain weight and width were significantly reduced compared to the wild-type but grain length was the same (Fig. 4). Starch content was significantly lower in mature grains of three RNAi lines whereas sucrose was increased, indicating that reduced starch content was not caused by limited sucrose in the grains.

**Fig. 5.**
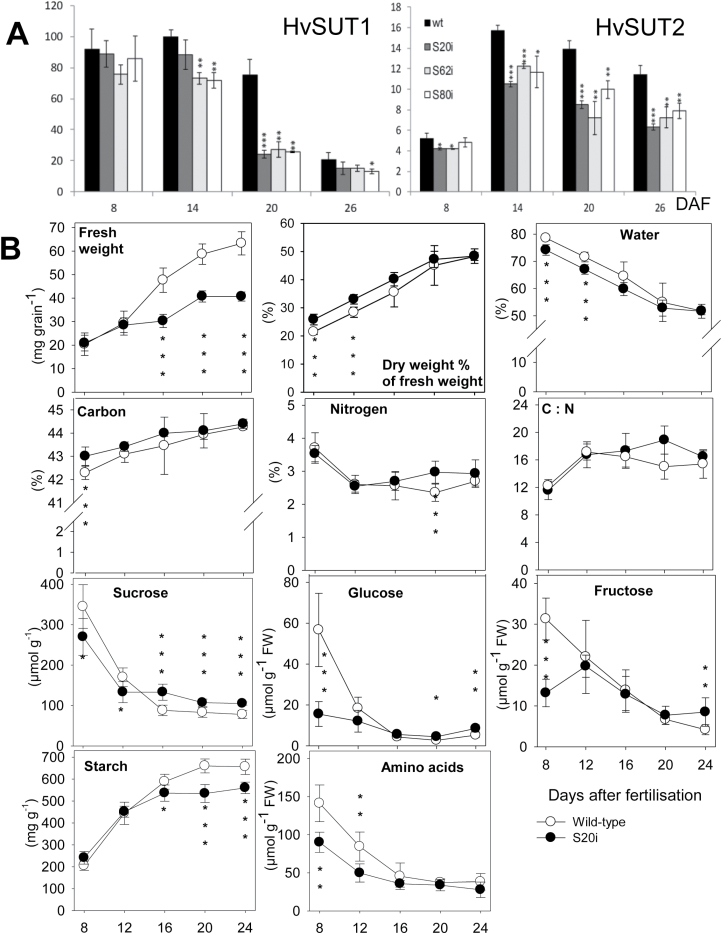
(A) Relative gene expression of *HvSUT1* and *HvSUT2* analysed in developing grains for the lines S20i, S62i, and S80i and the wild-type. (B) Fresh and dry weight, water content, starch and soluble components in developing grains of the S20i line and the wild-type. Data are means ±SD, *n*=3. Significant differences were determined by *t*-tests: *P<0.05, **P<0.01, ***P<0.001.

*HvSUT2* transcript levels were decreased in the three transgenic lines at all stages of grain development, with the strongest reduction between 14 and 25 DAF (Fig. 5A). *HvSUT1* expression was also decreased in all the analysed lines from 14 DAF onwards and followed the pattern of *HvSUT2* repression (Fig. 5A). The results showed that down-regulation of *HvSUT2* negatively affected *HvSUT1* expression not only in leaves but also in grains.

Transgenic S20i grains accumulated 50% less fresh weight between 16 and 24 DAF compared with the wild-type. However, dry weight was higher in the S20i line between 8 and 12 DAF and the grains contained less water at those stages (Fig. 5B). Whereas accumulation of total carbon was not significantly different, nitrogen content was higher in S20i grains during the late filling stage. Sucrose levels were decreased between 8 and 12 DAF but increased during later grain development. Similarly, glucose and fructose levels were decreased at 8 DAF but increased at 20 and 24 DAF for glucose and at 24 DAF for fructose. Starch levels were the same as in the wild-type until 16 DAF but were lower during later development in S20i grains (Fig. 5B). Amino acid concentrations were lower in S20i grains at 8 and 12 DAF but thereafter were the same as in the wild-type. Starch and free sugars were also determined in grains of lines S12i and S80i with similar results (Supplementary Fig. S2). In summary, transgenic grains were affected in dry matter and starch accumulation. Free sugars and amino acids were increased, especially at grain filling.

### Analysis of global gene expression in developing grains

Differential gene expression was analysed in the endosperm fractions of S20i and wild-type-grains at 2, 4, 8, 12, 16, and 20 DAF using the 12K barley macro-array (Sreenivasulu *et al.*, 2006). Genes were regarded as differentially expressed when significant changes of at least two-fold were observed in at least one stage (*P*<0.05, two biological replicates with two technical replicates each), and 822 and 704 genes were found to be up- and down-regulated, respectively (Supplementary Table S2). Differentially expressed genes were annotated and classified based on homology and a literature search. Our statements regarding gene identity and function should be considered as putative.

### Transcripts of starch and primary carbohydrate metabolism

**Fig. 6.**
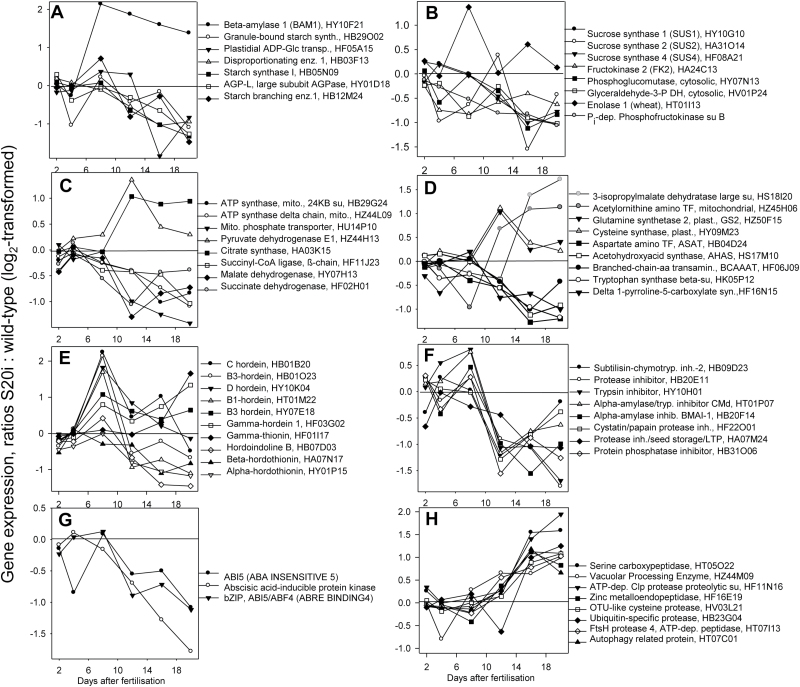
Ratios of gene expression in the endosperm fraction, S20i/wild-type, log_2_-transformed. (A) Starch metabolism, (B) sucrose cleavage and glycolysis, (C) mitochondrial metabolism, (D) amino acid metabolism, (F) grain maturation, (G) vacuolar transport, and (H) proteolysis.

All analysed genes associated with starch biosynthesis were down-regulated in grains from S20i plants during grain filling from 12 to 20 DAF (Fig. 6A, Supplementary Table S2), including *granule-bound starch synthase*, *starch branching enzyme*, *starch synthase*, *ADP-Glc pyrophosphorylase-L*, *disproportionating enzyme*, and plastidial *ADP-Glc transporter*. In contrast, *β-amylase 1* was strongly up-regulated between 8 and 20 DAF in the S20i grains.

Glycolysis was generally down-regulated transcriptionally in S20i grains, especially at 16 to 20 DAF, at the time of later filling involving *sucrose synthases 1*, *2*, and *4*, *fructokinase-2, phospho-glucomutase*, *PP*_*i*_*-phosphofructokinase*, and *glyceraldehyde-3-P dehydrogenase*. Interestingly, *enolase-1* was significantly up-regulated at all stages (Fig. 6B, Supplementary Table S2).

Three TCA-cycle genes were consistently down-regulated at mid-to-late grain filling, *succinyl-CoA-ligase*, *malate dehydrogenase*, and *succinate dehydrogenase*, together with other mitochondria-related genes such as *ATP-synthase*, *phosphate transporter*, and *carbamoyl-phosphate synthase*. Up-regulated expression of TCA-cycle genes included mitochondrial metabolism, *pyruvate dehydrogenase*, *citrate synthase*, *ATP-citrate-lyase*, and *mitochondrial solute carriers* (Fig. 6C, Supplementary Table S2).

The results indicated that grains in S20i plants displayed altered gene expression related to starch and primary carbon metabolism predominantly at mid- and later grain filling. Whereas sucrose degradation, starch biosynthesis, and glycolysis were down-regulated, starch degradation, enolase, and entry into the TCA-cycle were up-regulated.

### Amino acid and storage protein metabolism and seed maturation

Amino acid metabolism was differentially regulated in S20i and wild-type plants at the transcriptional level during mid-to-late grain filling. *Aspartate aminotransferase*, *acetohydroxyacid synthase*, *branched-chain-amino-acid transaminase*, *tryptophan synthase*, and *Δ-1-pyrroline-5-carboxylate synthetase* were down-regulated. *Acetylornithine aminotransferase*, *3-isopropylmalate dehydratase*, *glutamine synthetase-2*, and *cysteine synthase* were up-regulated (Fig. 6D). Storage protein gene expression was the same between grains of S20i and wild-type plants at early development. However, at 8 DAF, expression of most of the *hordein*, *thionin*, and *indoline* genes was transiently increased, following down-regulation at mid-to-late grain filling. On the other hand, several storage protein-related transcripts remained the same or were even increased in S20i grains at later grain filling (Fig. 6E, Supplementary Table S2). These results indicated that amino acid metabolism and storage protein synthesis were partially down-regulated at transcript levels at later maturation.

Several genes involved in seed maturation were down-regulated, especially during grain filling from 10 DAF onwards, including inhibitors of *subtilisin*, *chymotrypsin*, *trypsin*, *α-amylase*, and *cystatin/papain*, and *seed storage/lipid transfer protein* (Fig. 6F). These genes were generally abscisic acid (ABA)-inducible and encode protective enzymes against pathogens and stress, which accumulate during maturation, potentially in an ABA-responsive manner (Mönke *et al.*, 2012). Hence, ABA signalling was transcriptionally down-regulated, as seen for transcription factor *ABI5* (*ABA INSENSITIVE 5*), *ABA-inducible protein kinase*, and *bZIP* transcription factor *ABI5/ABF4* (*ABRE BINDING-4*) (Fig. 6G).

Some proteases displayed inverse profiles, being up-regulated in S20i grains from 10 DAF onwards (Fig. 6H). These included *vacuolar processing enzyme*, *Zn metallo-endopeptidase FtsH*, and *Clp proteases*, *autophagy-related protein*, and *proteases* involved in the ubiquitin pathway (Fig. 6H).

### Gene expression of transport-related proteins

**Fig. 7.**
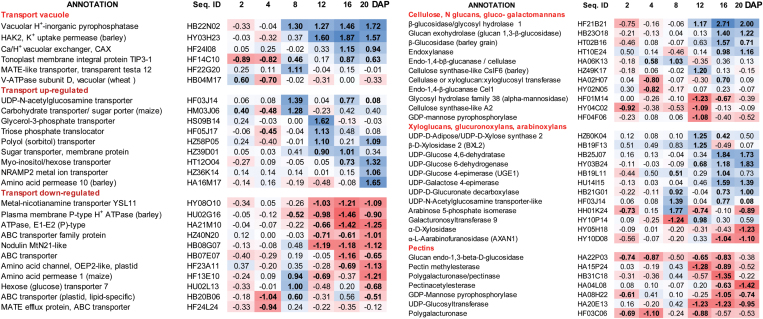
Ratios of gene expression in the endosperm fraction, S20i/wild-type, log_2_-transformed. Bold type indicates significant differences (*t*-test, *P*<0.05). Colour coding: red, down-regulated and blue, up-regulated in S20i with respect to the wild-type.

Gene expression related to vacuolar transport was also affected in S20i plants relative to the wild-type (Fig. 7). Energisation of vacuolar transport occurs via H^+^-inorganic pyrophosphatases and V-ATPases. In S20i grains, only the former was strongly up-regulated between 8 and 20 DAF. *HvHAK2*, a low-affinity Na^+^-sensitive K^+^ vacuolar uptake permease (Senn *et al.*, 2001) was up-regulated between 12 and 20 DAF. *Ca/H*^*+*^*vacuolar-exchanger* (*CAX-like*) and *tonoplast-intrinsic-protein* (*TIP3-1*) were increased at 16 and 20 DAF. Other membrane transporters were up-regulated predominantly during maturation from 8 to 20 DAF (Fig. 7), among them *UDP-N-acetylglucosamine transporter*, which is potentially involved in transferring cell wall components, *triose-P translocator*, *HvAAP10-amino acid permease*, and *NRAMP2 metal transporter*. Down-regulated forms during maturation were *metal-nicotianamine transporter YSL11*, *P-type H*^*+*^*-ATPase*, *OEP2-like amino acid channel*, and four *ABC transporters* with unknown functions (Fig. 7).

### Cell wall biosynthesis

Expression of several genes of cell wall metabolism was changed in S20i plants compared with the wild-type. Out of nine genes involved in the metabolism of cellulose, N glucans, gluco- and galactomannans, five were up-regulated and four down-regulated (Fig. 7). Genes involved in the biosynthesis of xyloglucans, glucuronoxylans, and arabinoxylans were mostly up-regulated, especially those involved in nucleotide (UDP) sugar metabolism. Remarkably, those transcripts involved in pectin biosynthesis were consistently decreased (Fig. 7). The significance of pectin in cereal endosperm is unclear, and whilst it is thought to be absent at least from mature barley endosperm it is present in wheat (Chateigner-Boutin *et al.*, 2014). The results revealed considerable deregulation of cell wall biosynthesis at the transcriptional level, predominantly during maturation of grains in S20i.

### Comparative metabolic profiling of developing grains

**Fig. 8.**
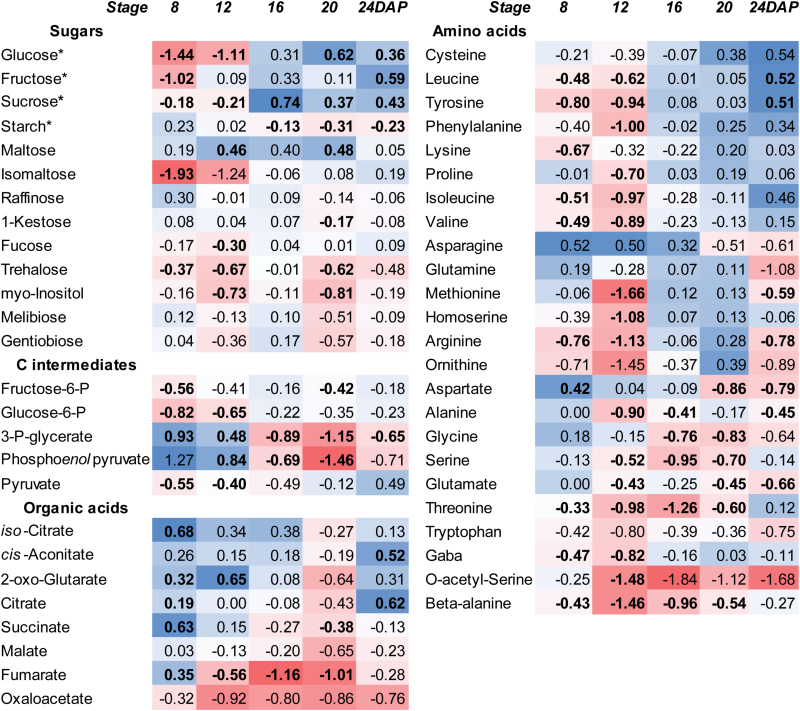
Changes in metabolites and amino acids in the endosperm fraction during grain development (ratios S20i/wild-type, log_2_-transformed), determined by GC-MS analysis. Colour coding indicates the abundance of metabolites: from dark blue for high ratio to dark red for low ratio. Data are means ±SE, and bold type indicates significant differences according to a Benjamini–Hochberg corrected *t*-test: *P*<0.05, *n*=8. *, values determined by spectrophotometric assay.

Comparative metabolite profiling was performed by GC-MS for S20i and wild-type grains at 8, 12, 16, and 20 DAF (Fig. 8). Levels of most free sugars and hexose-phosphates were decreased in S20i grains during early-to-mid development (8, 12 DAF) and increased (sucrose) or remained the same as in the wild-type during later grain filling. Levels of glycolytic intermediates of 3-P-glycerate and PEP were increased in S20i grains at 8 and 12 DAF but decreased or remained the same as in the wild-type (pyruvate) during later development. Whereas starch content was lower in RNAi grains between 16 and 24 DAF (Fig. 4), maltose levels were increased throughout development (significantly only at 20 DAF). Levels of the organic acids citrate, aconitate, isocitrate, and 2-oxoglutarate tended to be higher in S20i whereas levels of malate, fumarate, and oxaloacetate were lower. Total amino acid contents were analysed by GC-MS (Fig. 8, Supplementary Table S3) and by UPLC (Supplementary Fig. S3), and were lower in S20i grains at 8 and 12 DAF but not different to the wild-type afterwards (Fig. 5B). Accordingly, levels of most individual amino acids were lower than in the wild-type at 8 and 12 DAF, except for Asn and Gln, which were the same as the wild-type, and for Gly, which was lower from 16 DAF onwards.

### Sucrose accumulation in transgenic grain organs along the sucrose delivery route

**Fig. 9.**
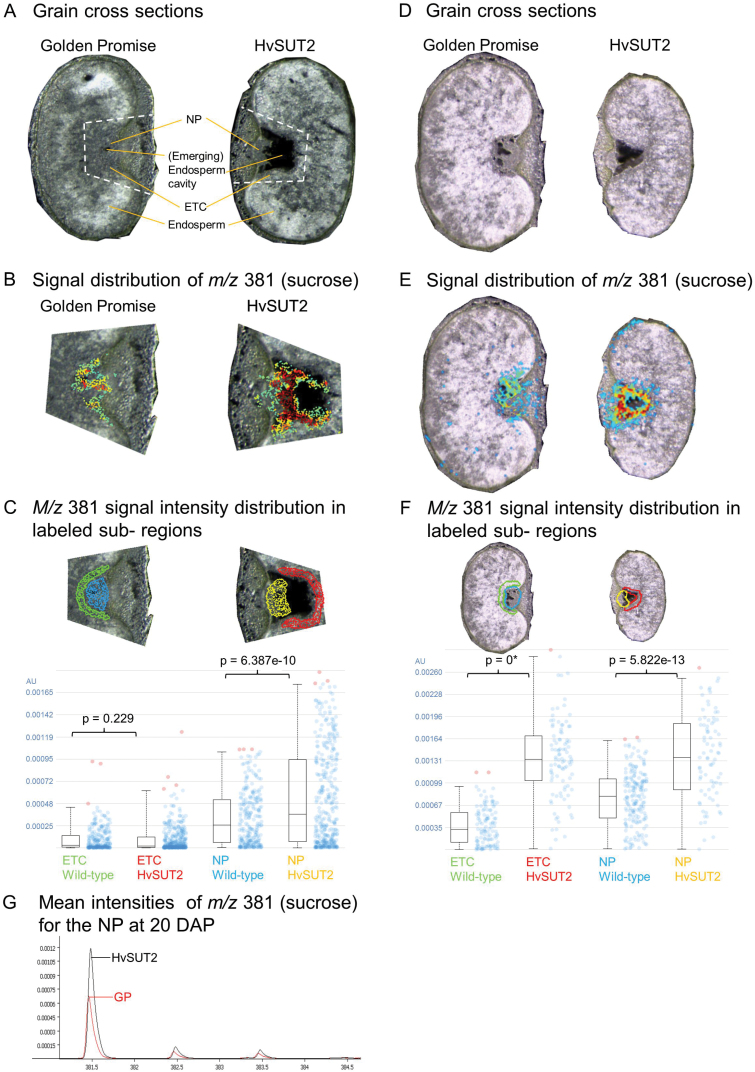
Sucrose accumulation patterns during mid-to-late grain filling in S20i and the wild-type, analysed by MALDI MSI. Histological images illustrate the developmental stages at (A) 14 d after fertilisation (DAF) and (D) 20 DAF; transfer tissues are indicated in (A). Sucrose distribution at (B) 14 and (E) 20 DAF for S20i and the wild-type. (C, F) Manually selected regions are illustrated and signal intensity distributions presented as box plots for the NP and ETC at 14 (C) and 20 DAF (F). Statistical differences between S20i and the wild-type are indicated. (G) Overlay of mean mass spectra for the NP regions from S20i and the wild-type grains at 20 DAF.

*HvSUT1* and *HvSUT2* gene expression overlapped temporally and spatially in the tissues along the assimilate transfer path, which consisted of vascular tissue, the NP, ETCs, and starchy endosperm cells (Fig. 2B). S20i endosperms seemingly became sucrose-limited during mid-to-late grain filling (Fig. 8), accumulating less starch and dry weight than the wild-type (Fig. 4) although overall sucrose and hexose levels were higher (Figs 4, 5, 8). We assumed that this was the result of suppressed sucrose efflux from vacuoles within the transfer path tissues, leading to trapping and accumulation of sucrose. To map its spatial distribution, mass spectrometry imaging (MSI) was applied on cryosections from S20i and wild-type grains at 14 and 20 DAF (Fig. 9A, D). When comparing intensities of the molecular ion *m/z* 381 (sucrose), the highest levels were observed in the NP and ETCs relative to the other tissues. Signal intensities were strongly increased in S20i compared to the wild-type (Fig. 9B, E). At 14 DAF, significantly higher levels of molecular ion *m/z* 381 were present in the NP of S20i, while levels in ETCs were the same as in the wild-type (Fig. 9C). At 20 DAF, levels of molecular ion *m/z* 381 were significantly higher in both the NP and ETCs of S20i grains (Fig. 9F). The overlay of mean mass spectra for the NP region at 20 DAF is illustrated in Fig. 9G, and shows higher intensities of molecular ion *m/z* 381 for S20i.

**Fig. 10.**
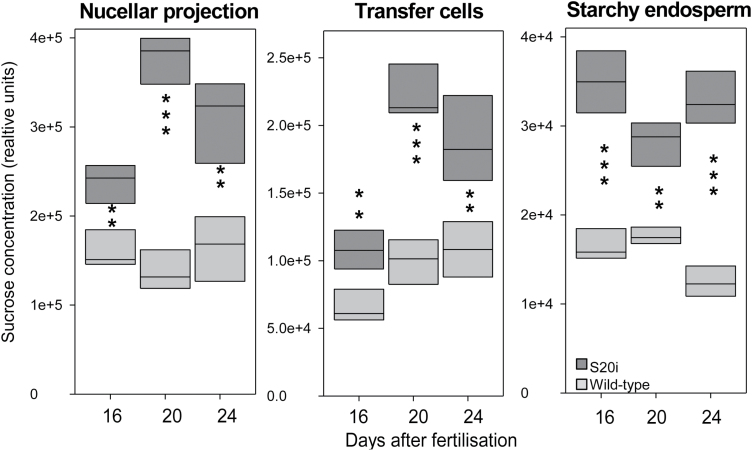
GC-MS analyses performed on micro-dissected slices of the NP, ETCs, and starchy endosperm at 16, 20, and 24 d after fertilisation. The box plots represent medians of eight repetitions, with significant differences as determined by *t*-tests, **, *P*<0.01, ***, *P*<0.001.

To further test sucrose concentrations in the organs of S20i and wild-type grains, GC-MS analysis was performed on micro-dissected slices of the NP, ETCs, and the central starchy endosperm at 16, 20, and 24 DAF. For all tissues and stages, sucrose concentration was significantly increased for S20i compared to the wild-type by 1.5- to 2.8-fold (Fig. 10). These and the MSI-derived results showed that sucrose accumulated in organs involved in sucrose transport all along the sucrose delivery route in transgenic grains.

## Discussion

In developing barley grains, plasma-membrane-localised sucrose transporter HvSUT1 and tonoplast-located HvSUT2 are co-expressed in the same grain cell types, obviously functioning in co-operation to control sucrose homeostasis. *HvSUT2* RNAi-repression negatively affects the expression of *HvSUT1* and reveals that concerted action of both sucrose transporters is required for grain filling and development. Although overall sucrose levels are higher along the sucrose delivery route, transgenic endosperm seemingly becomes sucrose-limited during mid-to-late grain filling, which strongly decreases starch levels. This suggests that HvSUT2 together with HvSUT1 controls sucrose balances during grain filling. When sucrose transport becomes deficient, specific sugar starvation responses are initiated, such as co-ordinated sugar recycling from starch, hemicelluloses, and celluloses, and vacuolar protein degradation. Genes involved in glycolysis, the TCA-cycle, and starch and amino acid synthesis are down-regulated, indicating that endosperm cells suppress certain pathways to conserve resources and to maintain important cell functions.

### Plasma-membrane and tonoplast SUTs are co-localised in grain tissues

Five sucrose transporter genes exist in barley (Fig. 1A), which is similar to rice (Aoki *et al.*, 2003). Expression levels and profiles reveal that HvSUT1 and HvSUT2 are particularly important for grain filling (Fig. 1C), and GFP experiments demonstrate that HvSUT1 is localised at the plasma-membranes and HvSUT2 at the tonoplast (Fig. 2A). HvSUT2 clusters together with the tonoplast-localised transporters OsSUT2, AtSUC4, and PtaSUT4 (Endler *et al.*, 2006; Eom *et al.*, 2011; Payyavula *et al.*, 2011; Schneider *et al.*, 2012), confirming HvSUT2 as a vacuolar sucrose transporter, which is consistent with its presence in the vacuolar proteome (Endler *et al.*, 2006).

During the pre-storage phase (2–4 DAF), both *HvSUT1* and *HvSUT2* were expressed mainly in the maternal grain parts along the assimilate transfer path, the vascular bundle and the NP. Compared to HvSUT1, HvSUT2 transcript levels were higher in early maternal grain parts. Cell type-specific expression was very similar for *HvSUT1* and *HvSUT2*, even though *HvSUT1* was expressed 10- to 100-fold higher in the endosperm and both transporters differed in timing of maximal expression, which was at early-to-mid grain filling for *HvSUT1* and mid-to-late for *HvSUT2*. Despite different sub-cellular location, expression patterns of *HvSUT1* and *HvSUT2* largely overlapped temporally and spatially during grain development, suggesting functional interactions during sucrose transfer and partitioning.

### Plasma-membrane and tonoplast SUTs function in a concerted manner

RNAi-mediated down-regulation of *HvSUT2* also generated *HvSUT1* repression, resulting in broader leaves in all three RNAi lines and higher levels of sucrose and starch compared to the wild-type. Similarly, poplar plants with repressed PtaSUT4 show elevated sucrose in leaves (Payyavula *et al.*, 2011). Since it is unlikely that the *HvSUT2*-RNAi construct also targets *HvSUT1* expression, we suggest that *HvSUT1* down-regulation is a consequence of *HvSUT2* deficiency. Since *HvSUT2*-RNAi expression is driven by the *HvSUT1* promoter, the strongest *HvSUT1* repression would be anticipated at the highest gene activities (e.g. at 8 DAF; Fig. 5A), when *HvSUT1* would be directly targeted by RNAi. However, we observed that *HvSUT1* down-regulation followed the *HvSUT2* repression levels, indicating that functional *HvSUT2* deficiency also decelerated *HvSUT1* gene activity.

Thus, co-regulation and functional interactions between both transporters is expected. The exact reason for the co-suppression of *HvSUT1* in the *HvSUT2*-RNAi plants is presently unclear. Since S20i grains obviously suffered from nutrient stress, this could cause down-regulation of *HvSUT1*. Similarly, in rice, *OsSUT1* expression is decreased by stress such as shading, which leads to sugar depletion (Ishibashi *et al.*, 2014). In poplar, repression of the vacuolar transporter *PtaSUT4* alters the ratio of transcripts of *PtaSUT3*, associated with apoplastic transport, to *PaSUT4*, associated with intracellular transport, indicating that reduced PaSUT4 can affect sucrose transport in various ways (Payyavula *et al.*, 2011). Interactions between co-expressed sucrose transporters have also been identified in Arabidopsis (Schulze *et al.*, 2003). Together, this suggests possible concerted actions of apoplastic and vacuolar sucrose transport in controlling cell sucrose homeostasis in barley grains.

### Sucrose transporter-repressed grains display starch deficient phenotypes

In all three RNAi-lines, grain dry weight was decreased and grain width was lower whereas length was unchanged relative to the wild-type (Fig. 4). Grains mainly grow by length during the pre-storage phase, 3–10 DAF, and thereafter they grow predominantly by width (Thiel *et al.*, 2012; Pielot *et al.*, 2015). Since *HvSUT2*-RNAi grains have reduced widths, *HvSUT2* repression is predominantly exercised when grains grow under filial control at mid-to-late grain filling, which is consent with reduced starch levels at that stage. Conclusively, *HvSUT2* suppression primarily affects endosperm filling rather than pre-storage development.

### HvSUT2 functions as controller of sucrose balances during grain filling

S20i grains accumulated 50% less fresh weight at 16–24 DAF than the wild-type. However, at early grain filling, 8–12 DAF, S20i grains had higher proportions of dry weight and contained less water. This could be explained by the fact that S20i grains at these stages contained lower levels of soluble components (sucrose, glucose, fructose, and free amino acids; Fig. 5B), which could affect the uptake of water. Obviously, during early grain filling assimilate accumulation in the endosperm is compromised by reduced supply from either vegetative organs and/or from the maternal grain due to down-regulated *HvSUT2* in these tissues. Accordingly, sucrose concentrations were higher in the NP of S20i at 14 DAF (Fig. 9). However, under our growth conditions this did not affect starch accumulation at 8 and 12 DAF. In contrast, at mid-to-late grain filling, starch and fresh weight accumulation were strongly reduced despite higher sucrose. This indicates that reduced starch synthesis was not due to limiting sucrose in S20i endosperms, but is rather it was related to the impaired channelling of sucrose from vacuoles to the cytosol. In fact, sucrose was higher in organs all along the delivery pathway: in the NP at 14, 16, 20, and 24 DAF, in ETCs at 16, 20, and 24 DAF, and in the starchy endosperm at 16, 20, and 24 DAF (Figs 9, 10).

### Differential gene expression reveals changed primary metabolism and sugar starvation

Differential gene expression in the developing endosperm of S20i and the wild-type reveals altered starch and primary carbon metabolism, particularly at mid-to-late grain filling. While starch biosynthesis was transcriptionally down-regulated, *β-amylase* was strongly up-regulated. This enzyme is normally synthesised at late grain filling, is associated with starch grains, and is important during germination (Hara-Nishimura *et al.*, 1986). Up-regulated *β-amylase* together with decreased starch and increased maltose levels suggested inhibition of starch biosynthesis and precocious starch degradation in S20i grains. Sucrose degradation and glycolysis were transcriptionally down-regulated and were accompanied by lower levels of glycolytic metabolites and hexose phosphates. *Enolase* was strongly up-regulated. It is potentially localised in vacuoles and up-regulated by stress, and could probably contribute to generate ATP via glycolysis to meet the demands of vacuolar H^+^-pumps for increased H^+^-driven transport (Shimaoka *et al.*, 2004; Barkla *et al.*, 2009).

Amino acid metabolism and storage protein biosynthesis were partially down-regulated at later maturation stages, and were accompanied by lower levels of most amino acids. However, some genes of amino acid metabolism were transcriptionally up-regulated, as a possible response to decreased amino acid pools by relief of feed-back inhibition (Binder *et al.*, 2007). TCA-cycle genes were mostly down-regulated, except for *pyruvate dehydrogenase* and *citrate synthase*, which catalyse entry into the cycle. Whereas TCA-intermediates from acetyl-CoA to 2-oxo-glutarate were increased, those from succinate to oxaloacetate were instead decreased. This indicates that in S20i endosperm cells the TCA-cycle mainly provides carbon skeletons for amino acid biosynthesis (Sweetlove *et al.*, 2010). Seed maturation genes were down-regulated, including those encoding protective enzymes against pathogens and stress, which are potentially ABA-inducible. Accordingly, ABA-signalling was also down-regulated, as indicated by the transcription factors *ABI5* (*ABA INSENSITIVE 5*) and *ABA-inducible protein kinase*, and the bZIP-transcription factor *ABI5/ABF4* (*ABRE BINDING-4*) (Fig. 6G). Sucrose and ABA are mutually related in signalling seed maturation. Sucrose levels affect either sensitivity or ABA levels and sugar signalling requires the ABA-transduction chain (Smeekens, 2000; Finkelstein and Gibson, 2002; Radchuk *et al.*, 2010).

Whereas seed maturation was constrained in S20i grains, autophagy and protein degradation and remobilisation events were transcriptionally induced (Fig. 6H). Vacuolar proteolytic activity is frequently induced by sugar starvation (Brouquisse *et al.*, 2007), and autophagy could also result from various stresses including starvation (Liu and Bassham, 2012).

Vacuoles are reservoirs for ions and metabolites that allow buffering of nutrient changes (Neuhaus, 2007). Vacuolar transport is energised by concerted actions of H^+^-V-PPase and V-ATPase, which create a proton-membrane force to energise transport against concentration or electrochemical gradients (Krebs *et al.*, 2010). Interestingly, in S20i endosperms, only H^+^-VPPase was up-regulated from 8 DAF onwards, suggesting its particular importance during grain filling to enhance the vacuolar H^+^-electrochemical gradient in response to repressed HvSUT2 activity.

Two other genes associated with vacuolar transport were up-regulated, *HvHAK2*, a potential tonoplast K^+^ transporter (Senn *et al.*, 2001), and *aquaporin-family member TIP3-1*, which forms channels to facilitate movement of water and small uncharged solutes (Kaldenhoff and Fischer, 2006). Up-regulation possibly adjusts ion homeostasis under stress in HvSUT2-deficient vacuoles. Five genes involved in nucleotide sugar biosynthesis and a *UDP-N-acetylglucosamine transporter* were up-regulated. Nucleotide sugars are universal glycosyl-donors to form polysaccharides, glycoproteins, proteoglycans, glycolipids, and glycosylated secondary metabolites, and are generated from UDP-glucose after sucrose cleavage by sucrose synthase and/or from storage carbohydrates (Bar-Peled and O’Neill, 2011). Concomitant to this, degradation of N-glucans (hemicellulose, cellulose) was stimulated, as seen by transcriptional up-regulation of five related genes, whereas N-glucan synthesis was down-regulated (three genes). This indicates altered cell wall metabolism.

It has been concluded that sucrose limitation in the cytosol due to sucrose transporter gene repression leads to N-glucan degradation, and hemicellulose and cellulose mobilisation (Contento *et al.*, 2004; Lee *et al.*, 2007; Mangelsen *et al.*, 2010).

## Conclusions

**Fig. 11.**
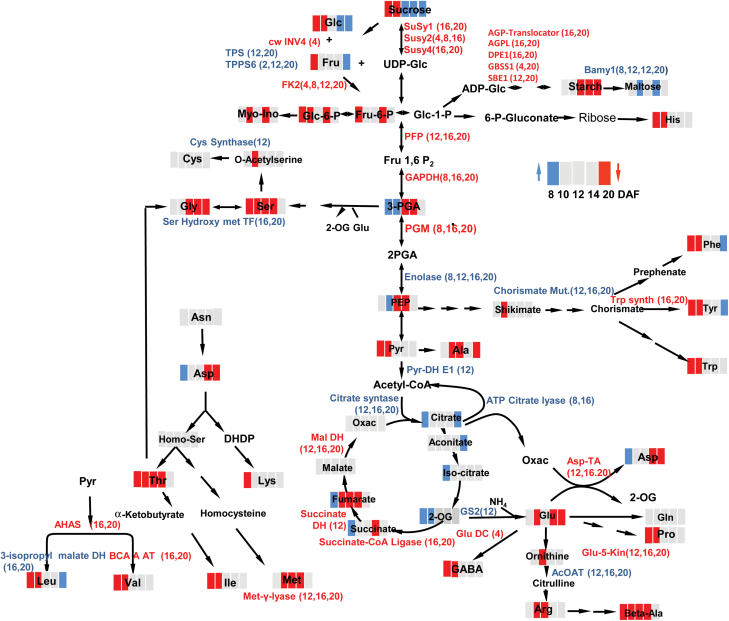
Summary of changes in transcript and metabolite levels in the endosperm fraction of transgenic S20i in relation to the wild-type. Data are derived from Fig. 6, Supplementary Table S2 (transcripts), Fig. 8 (metabolites), and Supplementary Fig. S1 (amino acids). Colour coding: red, down-regulated and blue, up-regulated in S20i with respect to the wild-type.

**Fig. 12.**
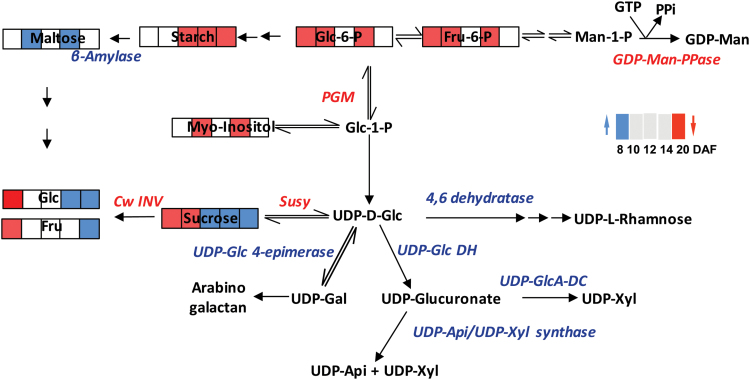
Integration of changes in metabolites and transcripts related to sugar, starch, and nucleotide-sugar metabolism into hypothetical pathways. Colour coding: red, down-regulated and blue, up-regulated iS20i endosperm with respect to the wild-type. Data are derived from Fig. 6, Supplementary Table S2 (transcripts), and Fig. 8 (metabolites).

In barley grains, *HvSUT1* and *HvSUT2* are expressed in the same tissues. Down-regulation of *HvSUT2* also reduces *HvSUT1* transcripts and generates leaf phenotypes that indicate co-regulation and functional interactions. In response to HvSUT2 deficiency, sucrose accumulates in organs along the transport route and within the endosperm. Nevertheless, the endosperm is seemingly sucrose-limited during grain filling and accumulates less starch and dry weight. This indicates suppressed sucrose efflux from vacuoles, leading to sucrose being trapped and accumulated in the vacuoles. HvSUT2 probably functions to control sucrose balances in the cytosol of the late endosperm, which is confirmed by high expression of *HvSUT2* at that stage. HvSUT2-repressed endosperm displays altered gene expression at mid- and later grain filling, with up-regulated sucrose degradation and entry into the TCA-cycle and down-regulated starch biosynthesis and glycolysis, and with lower levels of most free amino acids. On the other hand, starch degradation is transcriptionally up-regulated (Fig. 11). Cytosolic sucrose limitation leads to changed cell wall biosynthesis at the transcriptional level, such as N-glucan degradation and hemicellulose and cellulose mobilisation. This could contribute either to energy supply under sucrose limitation and/or to sugar salvage and formation of nucleotide sugars (Fig. 12). Changed cell wall and sugar metabolism and induction of sugar recycling from cell wall components most probably act to counter sucrose starvation effects. Barley endosperm can thus maintain basic levels of nucleotide sugar production under sucrose limitation.

## Supplementary data

Supplementary data are available at *JXB* online.

Table S1. Primers used in PCR and quantitative RT-PCR analyses.

Table S2. Differentially expressed genes in developing endosperm fraction of grains of line S20i.

Table S3. List of assigned metabolites (GC-MS) with corrected *P*-values.

Fig. S1. Comparison of the *HvSUT2* sequence fragment used for RNAi with the corresponding fragment of *HvSUT1*.

Fig. S2. Starch and free sugars in developing grains of lines S12i and S80i.

Fig. S3. Absolute amino acid contents determined by UPLC in endosperm of S20i and the wild-type.

## Supplementary Material

supplementary_table_S1

supplementary_figures_S1_S3

supplementary_table_S2

supplementary_table_S3
