# Supplementary material for: Down-regulation of the sucrose transporters HvSUT1 and HvSUT2 affects sucrose homeostasis along its delivery path in barley grains
Source: J Exp Bot. 2017 Aug 24;68(16):4595–612. doi: 10.1093/jxb/erx266 (PMC5853522; doi:10.1093/jxb/erx266)
Supplement: supplementary_table_S1 [file erx266_suppl_supplementary_table_s1.pdf]

Table S1. Primers used in PCR and quantitative RT-PCR analyses

Gene	Primer name	Sequence	Product size
Primers for cloning of *HvSUT3* and *HvSUT4* cDNAs			
HvSUT3	SUT3orf-dir	5’-AGCGCCCACTTGTCTTCCCGTA-3’	1681
	SUT3orf-rev	5’-GATCACTTTTGCAACCCAAGTTCCTG-3’	
HvSUT4	SUT4orf-dir	5’-ACCTAATCCCCGGCCTACCTCCTC-3’	1905
	SUT4orf-rev	5’-TGGGGATGAGGGTTTAGTTGTGTTCAG-3’	
Primers used for preparation of p1SUT2i construct			
HvSUT1	SUT1prom-Xho I	5’-CATCTCGAGATATCCCATTCCTATGATAAG-3’*	1445
promoter	SUT1prom-Spe I	5’-CACCGAGAGCTCCAACTAGTCGTTGC-3’*	
HvSUT2 sense	SUT2-Spe I-s	5’-CTGCTGACTAGTTACGTCCAGGAGCTGG-3’*	470
fragment	SUT2-Bgl II-s	5’-GGAAGATCTTGTACCAGCCATTGTACG-3’*	
HvSUT2 anti-	SUT2-Sal I-as	5‘-CTGTCGACCCCGTACGTCCAGGAGCTGG-3’*	470
sense fragment	SUT2-Xba I-as	5‘-GGAATATCTAGAACCAGCCATTGTACG-3’*	
Primers for qRT-PCR			
HvSUT1	qSUT1-d1	5’-GGCGGCCATCTGCATCATAAGC	112
	qSUT1-r1	5’-GGCGAAGAGGGCGAGGGAGAC	
HvSUT2	qSUT2-d1	5’-GGGAACGCTGCCTACACCACAC	145
	qSUT2-r1	5’-GGGCCCACGGAGGAAAGTAACA	
HvSUT3	qSUT3-d1	5’-GAGAGCCCCGGCAGGAACTTG	119
	qSUT3-r1	5’-AACGGCGGTGGAAATGAAAACA	
HvSUT4	qSUT4-d1	5’-ATCCGCGACGCCGAGACG	151
	qSUT4-r1	5’-GCAGCGCCCAGCCGAACT	
*Actin***	actin_u	5’-CTGGTTTCGCTGGAGATGATGC-3’	62
	actin_r	5’-CCGAGGGCGACCAACTATGC-3’	

* Restriction sites for fragment sub-cloning are underlined.

** *Hordeum vulgare actin* gene (Acc. Nr. AY145451).
