# Supplementary material for: Down-regulation of the sucrose transporters HvSUT1 and HvSUT2 affects sucrose homeostasis along its delivery path in barley grains
Source: J Exp Bot. 2017 Aug 24;68(16):4595–612. doi: 10.1093/jxb/erx266 (PMC5853522; doi:10.1093/jxb/erx266)
Supplement: supplementary_figures_S1_S3 [file erx266_suppl_supplementary_figures_s1_s3.pptx]

## Slide 1
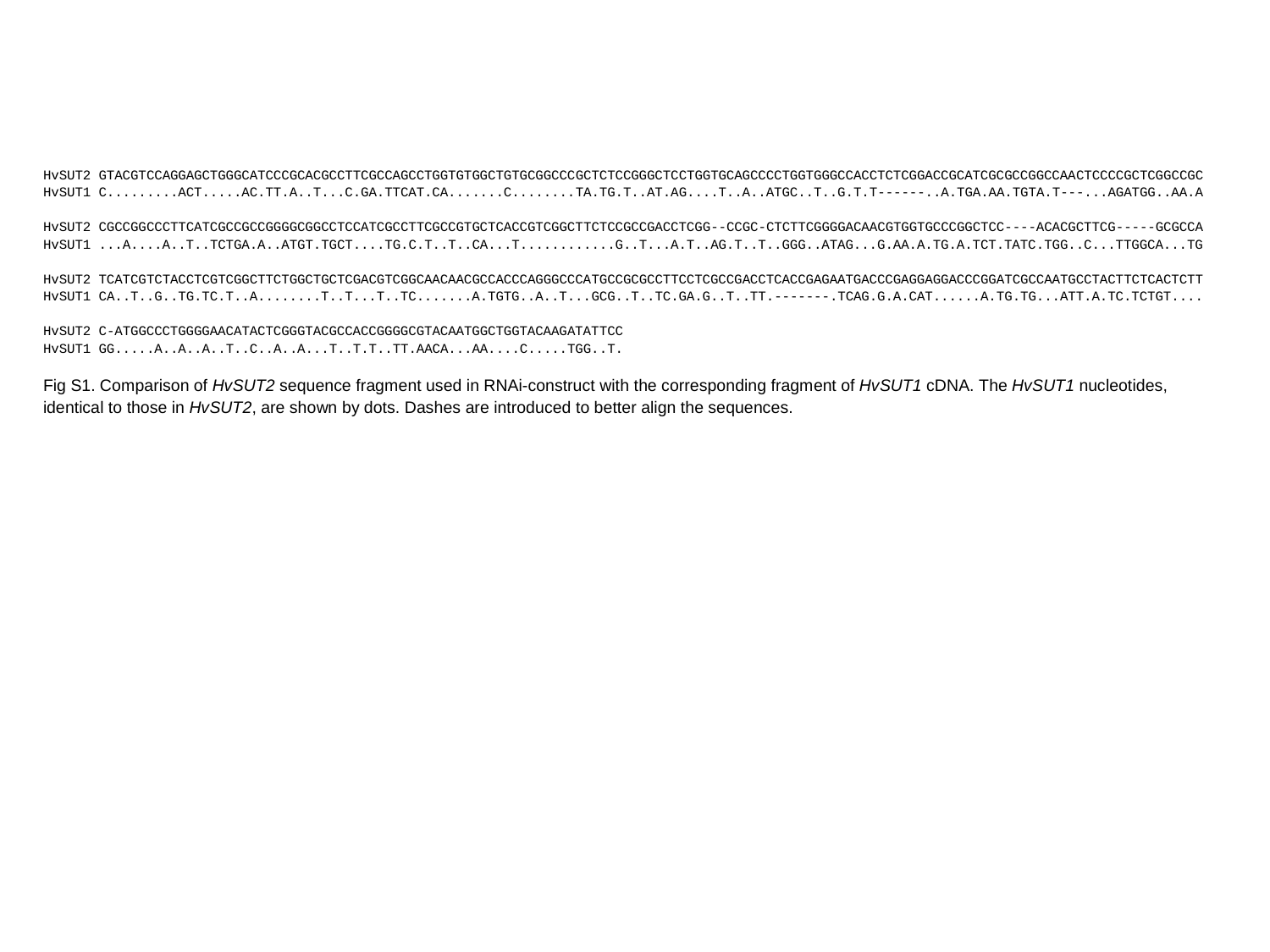

## Slide 2
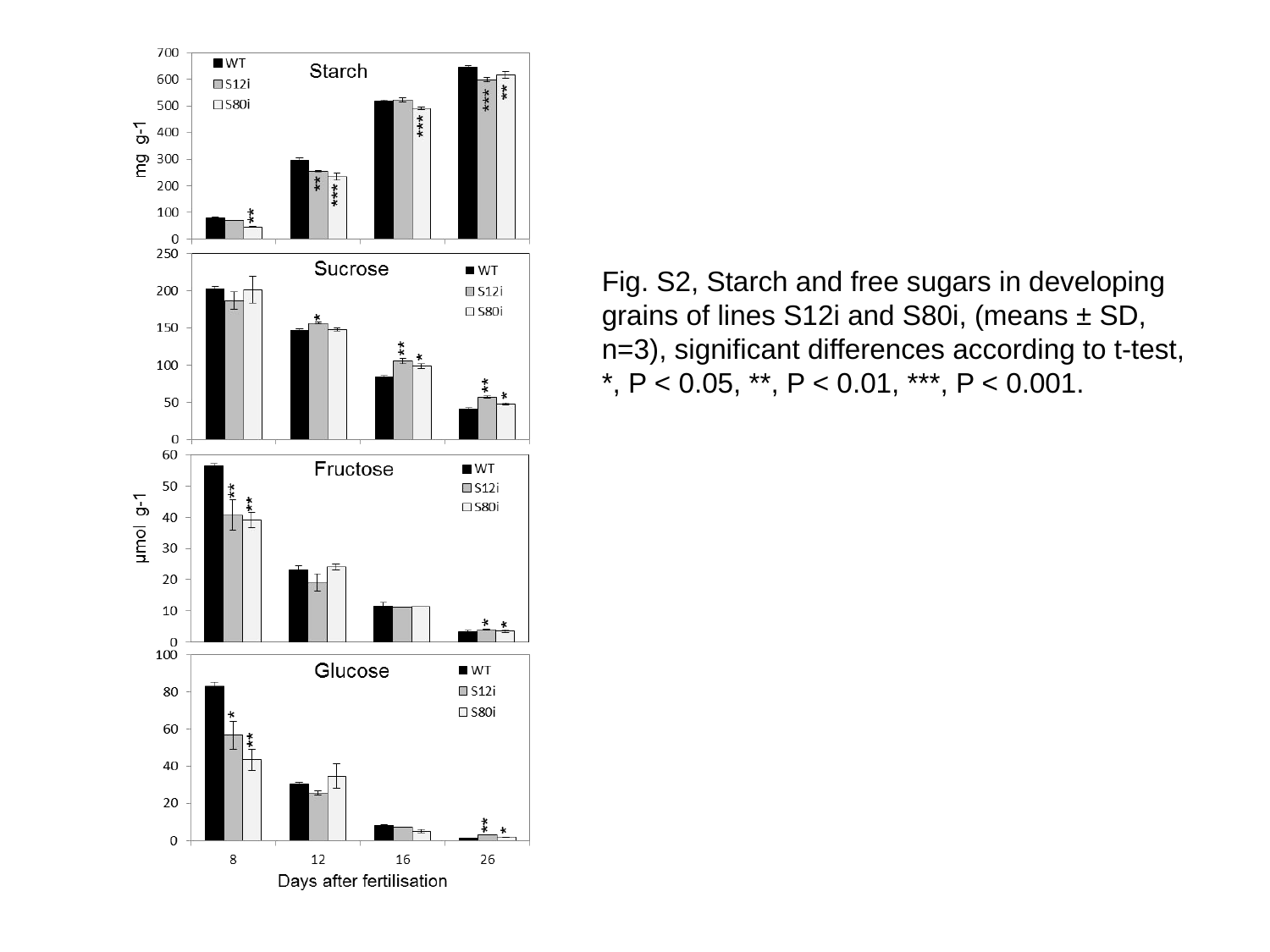

Fig. S2, Starch and free sugars in developing grains of lines S12i and S80i, (means ± SD, n=3), significant differences according to t-test, *, P < 0.05, **, P < 0.01, ***, P < 0.001.

## Slide 3
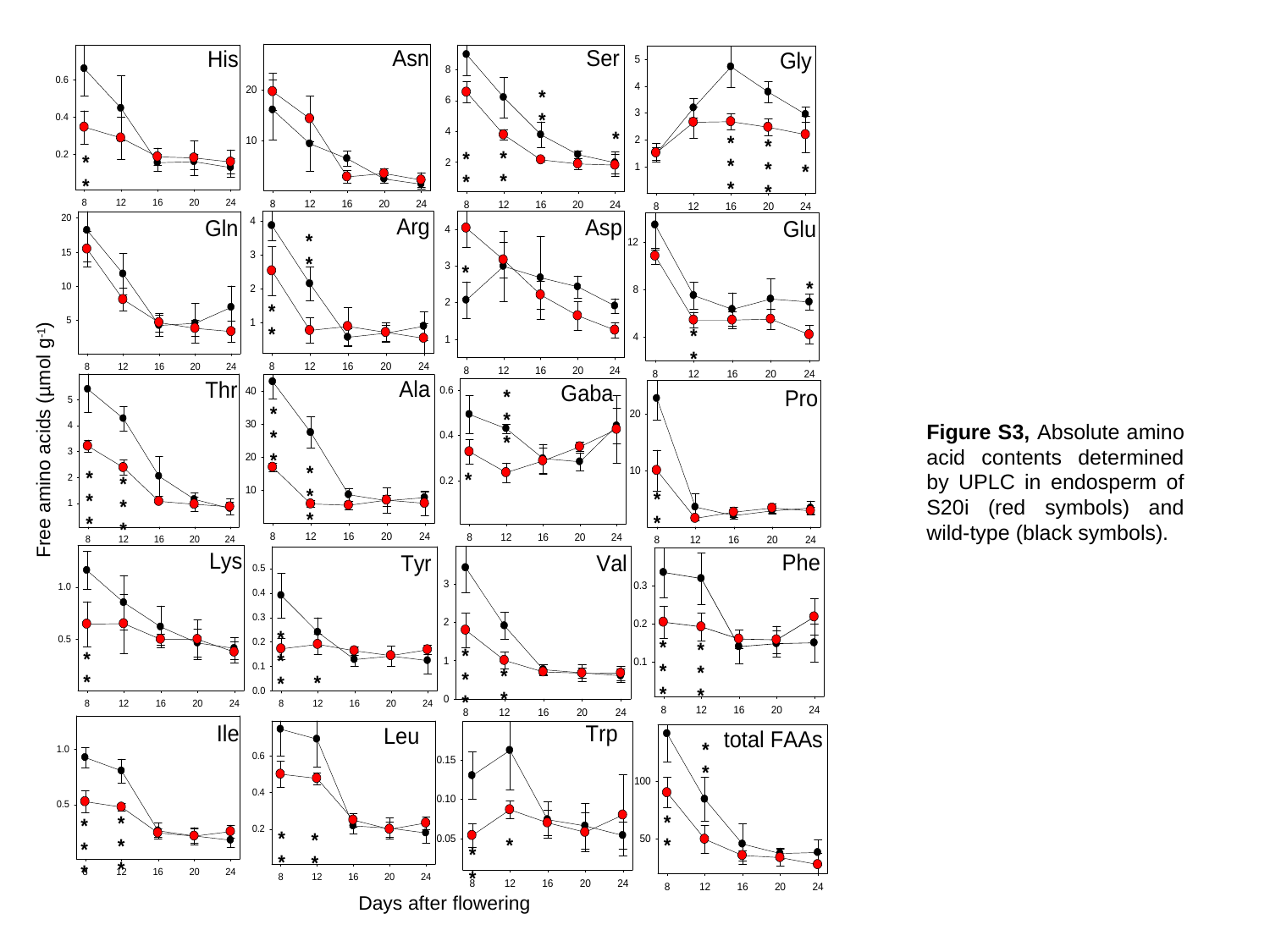

Figure S3, Absolute amino acid contents determined by UPLC in endosperm of S20i (red symbols) and wild-type (black symbols).
Free amino acids (µmol g-1)
Days after flowering
